# Living as an infertile woman: the case of southern and northern Ghana

**DOI:** 10.1186/s12978-020-00920-z

**Published:** 2020-05-20

**Authors:** Dorcas Ofosu-Budu, Vilma Hanninen

**Affiliations:** Department of Social Science, University of Eastern Finland, Kuopio, Finland

**Keywords:** Ghana, Infertility, Stigma, Women, Sub-Saharan Africa, Qualitative

## Abstract

**Background:**

Infertility is detrimental to the health of married couples, especially women. Despite the consequences associated with the condition, little is done to reduce the repercussions. This study throws more light on the lived experiences of infertile women and on how they can be helped to improve their own condition.

**Methodology:**

We interviewed 30 infertile married women in the Northern and Ashanti regions of Ghana, 15 from each region. A qualitative method, phenomenological study design and thematic analysis was employed to explore their lived experiences.

**Results:**

Due the huge stigma, some women reported that their loved ones encouraged them to try to conceive. Others revealed that, they were considering relocating from their current communities to reduce the insults, intense pressure, stress, and stigma. Though some revealed maltreatment from their husbands and in-laws, others stated their husbands disclosed their fertility status to their own family members to avoid these families pressuring these women.

**Conclusions:**

To reduce the stigma, pronatalist societies urgently need education and sensitization. Would-be couples should be counselled to have a plan to deal with such occurrences should they experience them.

## Plain English summary

Infertile couples have to deal with much societal pressure and stigma, especially in societies that take pride in having children. In such societies, women are deemed responsible for the situation. In trying to find causes for their condition, society claims that these women have been cursed or bewitched, that they might have committed abortions or have questionable lifestyles. Hence, though a challenging condition for couples, infertility makes the women suffer more.

This study, therefore, sought to find out the lived experiences of infertile women in Northern and Ashanti regions of Ghana. We explored how they are treated or perceived by family, in-laws, friends, and their communities.

We interviewed 30 women in total, 15 each from the two regions. The women reported the discomfort and challenges they faced. Some intended to relocate from their current communities to avoid the pressure and stress. Some stated even their husbands ridiculed them; others revealed their husbands were supportive.

Public education is necessary to reduce this burden on these women. People who are yet to marry should be counselled on how to deal with such situations should they occur.

## Background

Infertility has been very challenging throughout history [1, 2]. Unsurprisingly, it is a global health issue [3]. Infertility or childlessness is a major problem and can cause marital instability [3, 4]. People marry for several reasons; in sub-Saharan Africa, the main reason is childbirth. Hence, some studies in Ghana report that couples who voluntarily decide not to have children are unlikely [5]. Children are associated with happiness and viewed as a form of ‘intergenerational social security’ [6]. In Africa, women are mostly the first to be blamed if couples are unable to have children; right from childhood, they are ‘culturally raised’ to accept responsibility for reproduction and infertility [7].

Living as an infertile person is very distressing, particularly for women, due to stigma [5, 8]. It is associated with mental health issues, such as depression, suicidal ideations, insults, lack of sexual desires, and even divorce [8, 9]. Furthermore, infertile people are labelled witches, and considered useless and hopeless [8]. Some are bodily harmed; they sustain bruises and experience other forms of domestic violence from their husbands [8, 10]. However, Unisa observes that educated women, and societies with proper gender related institutional frameworks experience less violence [10].

Studies report that some infertile women share their predicaments with others for support, while some prefer to harbour the pain or share it only with their spouses [5]. This is because on disclosing their fears or challenges their image can worsen in the sight of others [5, 11]. Furthermore, in a study to investigate the strategies of women seeking infertility treatment in Southern Ghana, they anticipated that some strategies employed by infertile women were more harmful [5]. Thus, the need to explore their experiences and ways of coping.

Social support is vital in the life of infertile people; hence, those who avoid sharing their fears or keep their challenges to themselves are emotionally unstable [5]. Though some women report that their husbands are a source of encouragement to them, they feared change should the condition persist [5]. Also, in a study that explored the psychological consequences of infertility by Sultan and Tahir, infertile couples experience higher levels of depression, anxiety and aggression, lower levels of self- esteem, marital and sexual dissatisfaction [9]. However, in some studies, infertile women suffer more, and consequently, experience more psychological problems [12]. For example, in a study titled ´´I am anxious and desperate´´: psychological experiences of women with infertility in the Greater Accra region, Ghana, it was concluded that infertile women express worry, loneliness and lack of concentration [12].

The scale, gravity, and devastating consequences of infertility on the well-being of infertile women, especially in sub Saharan Africa, warrants the study of such women. Such studies can yield a better understanding of their situation and how best to improve their well-being.

### Aim of the study

We aim to explore the consequences of infertility.

### Theoretical underpinnings

As a theoretical background, we employed Goffman’s theory of stigma. According to the theory, stigma is a deeply discrediting attribute which reduces a whole person to a tainted and discounted other [13]. Stigma has negative connotations, inferior status, and relative powerlessness; that society collectively accords people who possess certain characteristics (or belong to a category) [14]. According to Goffman, stigma can be put into three categories, namely, physical differences, perceived character deficiencies and tribal stigma of race, nation or religion [13]. In all of these categories, the victims are socially perceived as unique from others, not human and hence disregarded [13]. Stigmas are either ascribed or achieved; thus, either born with or attained [15]. Stigmas are not inherent weaknesses in character or body but a social label, created by others in a society [15].

In relation to infertility, the victims belong to two categories; thus, are physically different and have perceived character deficiencies because of the causes and meanings attributed to infertility by society. For instance, in most pronatalist societies, infertility is perceived to be the result of promiscuity and abortions [16, 17], hence, character of the victims are questionable. More so, others attribute infertility to a curse, being bewitched or a biological cause [18]. Therefore, others avoid them to escape the wrath of deities responsible for their predicament. Further, since some infertile women report of irregular menstrual cycles, hormonal imbalance, unexplained causes and problems in their womb, they are perceived as incomplete or deformed. Consequently, though the women may not necessarily be physically deformed, they are still seen as deformed. This is because, to others, a complete woman should bear children.

Due to the consequences of stigma, sufferers try to conceal their conditions, to avoid it. In some instances, like blindness, and other physical disabilities the conditions are visible; hence, the sufferers are unable to conceal. However, while those with invisible conditions try to conceal it easily, in certain cases, it is impossible. For instance, though infertility may not be visible to onlookers, in pronatalist societies, due to the value and significance placed on children, infertility is socially and culturally visible.

Every society has laid down rules and regulations embedded in their culture; these norms regulate their way of life as well as control their attitude and behaviour [19]. Any deviation from these norms are frowned upon by members of that society. For example, generally in sub-Saharan Africa, society expects girls at a certain age to marry and have children. Therefore, childlessness in marriage is a challenging situation associated with curses, witchcraft, abortions, and other unhealthy lifestyles [8, 18]. Hence, infertile couples, especially such women, are perceived as different, inferior, and discounted. Among the Akans of Southern Ghana, which is also a study area of this research, there is a popular proverb which says that “a barren woman is synonymous to a landless person”. This emanates from the fact that land is the main source of production in “agrarian societies”. These and other socially constructed attributes of stigma adds to their predicaments, making them feel unworthy and hopeless. This situation stems from the labels, perceptions, and circumstances, society ascribes to this condition. Goffman also states that society forces one to go back-and-forth between different complicated roles, and consequently becoming untruthful, inconsistent and disreputable. This explains why the women have devised many ways of coping; with some pretending to have children just to avoid intense stigma and to appear socially acceptable.

## Method

### Study design

The phenomenon under study is very private and sensitive; hence, the need for a qualitative personal in-depth interaction is paramount. Specifically, phenomenological study design was employed to clarify and explore the lived experiences of infertile women. To better reveal and understand the lived experiences of involuntarily childless women, we used qualitative interviews with first-hand experience of this condition and thematic analysis aimed at exploring the descriptions and treatment of, and explanations for infertility among these women, as well as the meanings they constructed from their conditions. This paper explores the consequences of living as infertile women and the meaning they constructed from their experiences.

### Ethical considerations

The ethics committees of the Universities of Eastern Finland and Ghana (Humanities section) granted us approval; verbal and written consent was obtained from the participants. Those who could not read opted for verbal consent; some of those who could read were more comfortable with verbal consent. In all, only two women in the Ashanti region gave a written consent. The researcher translated the consent form into participants´ preferred languages. A cover sheet containing demographic information except for names was coded differently to ensure confidentiality. Specific participant locations are unreported; reporting could lead to easy identification.

### Study areas

The study areas were the Northern and Ashanti regions of Ghana. To capture the traditional and cultural contexts of the phenomenon, we combined the East and West Mamprusi Districts to form the northern location; and Kwabre East and Kumasi Metropolitan assembly to form the southern location (Ashanti region). East Mamprusi is located in the north-eastern part of the Northern region, sharing its west boundary with West Mamprusi [20]. The people in the Northern region speak the Mampruli language. Almost all the inhabitants in the Northern region are Muslims and in polygamous marriages. Polygamy is a common practice for members of the Islamic community and those who align themselves with African Traditional Religion [21]. According to the Akan culture, of which Ashanti region is a part, children inherit property from their mothers but not from their fathers.

### Ghanaian culture

Two main family systems exist in terms of inheritance [22]. Southerners mostly practise the matrilineal family system, except the Akuapims, Gas, and the Ewes, while the Northerners practise the patrilineal family system [23]. In these societies, children are a source of wealth and prestige to parents, especially older parents [24, 25]. Chiefs are installed in a process termed “enskinning” in the North and “enstooling” in the South among the Akans [23, 26]. ´´Enskinning ´´or ´´enstooling ´´is a ritual process by which a person is made king depending on their geographical location [27]. Thus, in the process of enskinment, the chief sits on a seat made of animal skin while in the enstoolment, the chief sits on a stool, made of wood. For the Akans, the days of the week are either male or female;, for prosperity, they marry only on female days: Tuesdays, Thursdays, and Saturdays [23].

Ghanaians recognise both monogamous and polygamous systems of marriage; most importantly, marriage is between two families and not merely two individuals [28]. Some studies highlight a direct relationship between one’s location, religion, and type of marriage [24]. For instance, polygamy is commonly practised by Muslims while Christians align themselves with monogamy [25]. A North-South divide exists; in the North, poverty is prevalent, and women are mostly dependent on their husbands [29].

### Herbalists as aid in recruitment

The herbalists were contacted with the help of stakeholders in the communities. The stakeholders were sub-chiefs and family heads in communities. The participants were recruited via their herbalists. The herbalists are people who have acquired knowledge and experience from treating with herbs all forms of ailments and conditions. They mostly acquired their experience from nature or family members over the generations; only one herbalist I met had formal education in herbal medicine. All these herbalists were middle-aged to old men. Although the use of orthodox medicine is common in Ghana, the use of herbal medicine is growing.

### Participant recruitment

In recruiting the participants, snowball technique was used. The first author informally contacted the sub-chiefs and family heads to help her identify the herbalists with whom the women visited for treatment. In the two geographic areas, the study and its purpose were discussed with the herbalists, who then informed their clients. These herbalists provided names and addresses of potential research participants; we later contacted and interviewed these participants in their homes. Some women shared with us the names of others with similar problems in the vicinity. In total, we interviewed 15 Muslim women aged between 19 and 41. They had been married for 4 to 16 years in the West and East Mamprusi District. In the Kwabre East and Kumasi Metropolitan assembly in the Ashanti region,15 women aged 21 to 43 participated. They comprised 13 Christians and 2 Muslims and had been married for 1 to 10 years. Participants hailed from different socioeconomic backgrounds.

### Data collection

The main data collection method was in-depth interviews. With 15 in each region, we interviewed 30. The interviews took place at the homes of the participants and under shade trees close to their homes where they considered comfortable. The principal investigator conducted all the interviews in the Ashanti region by herself; but for the northern interviews, she had the help of a translator. To make the participants feel comfortable, a considerable amount of time was spent in establishing rapport; they then felt free to express their concerns and grievances. This rapport was established before the interviews commenced and helped ease the tension in terms of class and cultural differences. The researcher was careful not to counsel or provide therapy until after the interview and only when it was deemed necessary.

The translator was both a native speaker of the language and a graduate teacher. In the Ashanti region, the researcher needed no translation; she is a native speaker of the language. The interviews were tape-recorded, with participants’ permission. Since the data from the north were already translated into English and recorded, they were typed and transcribed by the Principal investigator. The researcher noted her observations as well as the demeanour, mood, and body language, of the participants, aspects recorders fail to capture. Each interview lasted 45 to 60 min.

### Data analysis

After listening to the interview recordings, we transcribed them verbatim; with Times New Roman font, a font size of 11, and 1.5 spacing, this document had 386 pages. The interviews from the Ashanti region were translated from Twi to English by the First Author; the interviews from the North were already in English. After we repeatedly read them, the texts underwent thematic analysis. From the transcripts, we extracted four main themes; we then proceeded to the sub-themes. The main themes were feelings as expressed by the infertile women, interaction in the social environment, discouraging comments from others, uplifting and encouraging words from others and coping with being infertile. In establishing themes, we extracted statements with meanings that emerged in most transcripts. The analysis followed the guidelines of the coding manual for qualitative researchers by Johnny Saldana [30].

### Findings

As a means of coping, some of the women wished to relocate to avoid pain and trouble from their in-laws and community. Some reported that their husbands were responsible for their childlessness; these women informed their families of this situation, to avoid undue pressure on themselves. Others revealed that even their husbands stigmatized them. Furthermore, women pretended to have children to avoid stigma among their friends. Due to the stigma linked with divorce and to maintain their respect and honour, some women preferred to remain in their marriages. Noteworthily, regardless of one’s contribution in society, people are viewed as worthless unless they had children.

### Feelings as expressed by the infertile women

Generally, women are perceived responsible for reproduction. Hence inability to fulfil that need leaves the women with discomfort, worry and feel incomplete. Therefore, their feelings in this instance is from their own perception and not resulting from stigma.

Some women felt sad whenever alone. According to them, had they had children, they would have kept them company. Hence, they find themselves pondering over their childlessness.

“*What makes me worried and get sad is when I am alone, and my husband is not around … I feel if I had a child, we would have been spending time together because the house is very big … when I am alone … and I ponder over it, I get sad and just ignore and watch television.*”

(a 43-year-old, Ashanti region)

Some infertile women report feeling sad because of the utterances of fellow women playing with their kids. Some depict those who have children as blessed.

“*sometimes, I become very quiet and really very sad … The kind of things she says while patting, adoring, and pampering her children or child will make you sad.*”

(a 26-year old, Ashanti region)

Some women report feeling sad and bad when they hear children calling their parents. This reminds them of their failure to have children.

“*When sometimes I am alone … and my husband and little girl (niece) are not around … I sometimes go and pick her from school when the driver is unable to do so … and … when … I hear children calling their parents … mummy, daddy … when they are picking them from school … I feel very bad.*”

(a 28-year-old, Ashanti region)

Marriage without children makes them feel worthless, useless and, in a sense, unmarried. In some cases, they feel it would be better for them to divorce and rather busy themselves with other things.

“*Marriage without children, you will look like … you are not part of the world or you are not even part of the marriage, so it’s better you leave and go and look for some job to do and take care of yourself and your sibling’s children.*”

(a 35-year-old, Northern region)

#### Interacting in the social environment

Married women can only be recognised as such by having children although they might be unable to cater for them; else, irrespective of their contribution to society, they are perceived as irresponsible or useless.

“*You are seen as very useless, and the only way for you to be useful is to give birth because people see those infertile women as useless people...they will even honour and praise you if give birth to six children and you cannot even afford to cater for one … people will hail you...as you have done well, but no matter what you have done or do in the community here … without one child … no one will respect you and won’t even include you in their affairs.*”

(a 35-year-old, Northern region)

Some women remain married because society perceived married women as worthy of honour.

“*I will not leave, and the reason is simple … being married gives you, the woman, a dignity … so as a woman...if you are not married...it means you do not have support anywhere … so I will remain in my husband’s home no matter the circumstance or suffering*”.

(a 40-year-old, Northern Region)

### Discouraging comments from other people

One intrinsic feature about infertile women is their predisposition of feeling inadequate and worried about their plight. Therefore, any insults rained on them, reminds them of their condition which triggers and aggravate their plight.

Some women grieve whenever they encounter insults. These insults, according to some, make them lose both appetite and sleep.

“*At any day that insults are rained on me or accusations or pointing fingers...I can’t sleep that night, so I have experienced several sleepless nights and sometimes, cannot even eat food.*”

(a 25-year-old, Northern region)

People speculate and pass comments on the reasons for someone’s inability to have children. They further suggest solutions; these comments sometimes come also from people who appear to care. Some of their utterances show they are deceitful.

“*Some people pass comments … they can say for example, there are stones in your stomach … and if you don’t go and remove the stones, you can never give birth … meaning you are sick, so if you don’t go and treat yourself, you can never give birth … people who claim to like you say those things to you, and others will also insult you indirectly, saying that you have placed things in your body … say family planning pills … so both men and women stigmatize (someone) especially when they are aggrieved with the person*”.

(a 23-year-old, Northern region)

Some infertile women are questioned why they keep working for money despite being childless. It appears abnormal or strange to others to see childless women work; they wonder how such women spend their money.

“*It’s obvious … in the market...someone who sits beside me in market … sometimes...tells me you are doing well...some of them pass comments that if it wasn’t for their children … what will be their purpose for coming to the market...to look for money for what … so whenever they want to insult me...they begin talking to me like that.*”

(a 40-year-old, Northern region)

Infertile people are always insulted and told to focus on having children and to exploit any opportunity to do so. They can easily become a victim of circumstance although they have no idea about the situation.

“*the insults are too many to recall … but just today, on the way here, I dropped at a friend’s house just to say hello...not knowing that my friend had had a quarrel with another lady in the house. So when I got there, the one who was quarrelling with my friend began insulting me … to mind my own business and that instead of me focusing or concentrating on getting a baby … I am rather coming to people to gossip and coming in to insult her … she continued saying … there is no way I can leave my mission of trying to have a baby and come here to insult her. Therefore, the only task for me is to have a baby, and as for her...she has had five children and going to have her sixth … so I should look for mine … and that has tormented me very much … just today … just this afternoon.*”

(a 32-year-old, Northern region)

Infertile women are stigmatized throughout their lives; it becomes their label for life:

“*It means once one cannot give birth at all, it becomes a very big insult throughout one’s life. There are some women here who could not give birth from the beginning to their old age, and they have no peace … they have always been insulted by all manner of people.*”

(a 23-year-old, Northern region)

Some women report insults from their fellow co-wives; the former is still married but have nothing to show for it.

“*According to my co-wife … She is enduring all the sufferings in the house because of her children … and for this reason, she is staying in the house...if her child does something … she says...to the child … I am coping in this marriage … else … I wouldn’t have been here … by implication … what am I waiting for in the marriage since I have no child?*”

(a 40-year-old, Northern region)

Interestingly, some women reveal that they are stigmatized even by their husbands, especially by those who have children with their other wives in polygamous marriages.

“*One day...he travelled, and upon his return … he bought a new motorbike, and early in the morning … when he went for prayers and came back … he took the motorbike and came outside, and he started calling my co-wife’s little boy … J … J … this is your daddy’s motorbike...your daddy bought it for you … he will be using it to take you around, and say if someone doesn’t like it … the person can burst (Sic).*”

(a 35-year-old, Northern region)

Reports exist of maltreatment from some in-laws. Some in-laws attempt to dump their childless daughters in-law just to please the co-wives who have children.

“*With the exception of one of the younger sisters of the husband who somehow consoles me, being a little closer … or friendly to me but the rest of the family … everyone sees me as useless … even at a point, the husband’s father (my father in-law) made an attempt to kick me out of the house simply because the second wife claims whenever she sees me … her heart beats arrhythmically … and her blood pressure rises, so she suspects I am likely to harm her in one way or the other, so they nearly kicked me out of the home of her husband.*”

(a 32-year-old, Northern region)

### Uplifting and encouraging words from others

The actions of other persons were not always negative, but still they carried the idea that infertility is something to overcome.

Some women had some family members advising them to dedicate time and effort to their search for children.

“*Right now, it is my mother who sometimes advises me to stop working and focus on getting a child.*”

(a 45-year-old, Ashanti region)

Some mothers advise their daughters to change partners in order to conceive; they feel their daughters could be lucky with a different partner.

“*My parents are advising me to quit … and look for someone else, and maybe things will be better … if I leave the relationship, I could also possibly have a baby from someone else, so I don’t think it’s advisable to remain in this relationship.*”

(a 34-year-old, Northern region)

Some friends and families keep asking about the situation. Though it can be disturbing, these well-wishers do so to show love and care**.**

“*It is not like they are pressurizing you … they are not … they are just like oh … so what are you doing about it?...are you doing something about it?....which indirectly shows that they are concerned, and you should give them some grandchildren to run around.*”

(a 29-year-old, Ashanti region)

Some women stated that their husbands informed their own families about the cause of their childlessness to avoid pressures on these women.

“*Some few months ago, my husband himself told them the problem, and I don’t know why he did that, and according to him, he didn’t want any pressure to be coming from his family … on me, so he just told them what the problem was, but he is also the type who wouldn’t just allow someone to put pressure even on him … we are just okay by ourselves … but according to him … in our local setting and in Ghana, people will surely be looking forward to a child, so he went forward to the family and informed them that the problem was his, so they should not worry me about anything … so that’s what he did*”.

(a 30-year-old, Ashanti region)

#### Coping with being infertility

There were different ways in which the women tried to cope with the situation, ranging from thoughts of relocation to finding places to relax. This could be conceptualized as ways of concealing the stigmatizing condition.

Due to insults or verbal abuse, some women preferred to relocate to avoid the pressure and discomfort.

“*I don’t have peace of mind, so when I go to the school, and it closes at 3:30pm, I usually stay back to relax because it is very uncomfortable at home...I leave the school at 5pm, apart from Tuesdays and Fridays, when I organize extra classes...so possibly, it’s either my husband understands the issue at hand for us to relocate to a different place, away from the family house, or I get pregnant.*”

(a 31-year-old, Ashanti region)

Some women stated that they kept their condition to themselves to avoid insults from others. To themselves, they do not appear as someone who can easily be insulted or humiliated based on their appearance or looks.

“*My dear, have a look at me … can you insult me? … I do not talk to people about it...so most people are not aware, for me to attract insults … my dear … even where I stay, I think only about two or three are aware.*”

(a 43-year-old, Ashanti region)

Interestingly, some women revealed that they tell their friends they had children just to avoid stigma.

“*even my close friends insult infertile women in my presence … saying instead of them having children, all they know is fashion because they do not know my story … and I have not told them because of the way they talk about infertile women, they think I have children … , so I tell them that I have three children and that they are on their own because they are grown up … I further mention what they do, etc … when people come around and leave, they say all kinds of things about the people … e.g....instead of focusing on having children … all she knows is fashion, so if they get to know … they will say the same about me though I hardly dress up … .the little I wear is seen as very nice.*”

(a 43-year-old, Ashanti region)

## Discussion

The results indicate that infertile women are highly stigmatized in Southern and Northern Ghana and are not recognised by society. This finding is in line with studies reporting that infertile couples are stigmatized and denied social status in their communities [29]. The study also corroborates existing knowledge in pronatalist societies, where childbirth is hailed and associated with good will and blessings, while childlessness is linked to curses and witchcraft [18] Additionally, most women reported a sense of worthlessness and uselessness; they experienced sleepless nights. This feature is consistent with studies that establish that infertility breeds psychological challenges affecting the well-being of couples, especially women [8, 9, 31]. We anticipated this result; societal pressures and stigma exacerbates the plight of these women, rendering them susceptible to stress, depression, and anxiety. Hess, Ross and Gilillard examined infertility-induced psychological distress and coping strategies among 58 infertile women in Mali. They used a convergent mixed method design, and 73% of the women stated that they were psychologically disturbed. The same study revealed that 23% of the women were criticised by in-laws. We also report that in-laws find fault with their daughters-in-law. In the current study, in-laws threaten to throw them out from their matrimonial home because their co-wives are uncomfortable seeing them around.

Some women stated that their rivals or co-wives quizzed them why they were still married when they had nothing to show for it. Their husbands also teased and insulted them through children from their co-wives, a phenomenon also reported by Hess and colleagues [31]. They established that infertile women experience tension in their marriages, especially with their husbands.

However, other husbands disclosed their fertility status to their own relatives to prevent them from pressurizing their wives; Donkor and Sandall report this feature. In their study exploring the coping strategies of women seeking infertility treatment in Southern Ghana, they revealed that some women receive support from their husbands [5].

Most women in the Northern region were unemployed housewives. They expressed their discomfort and stress; their husbands no longer provided for them as they had done previously. This is buttressed by Fledderjohann [8]. Although both studies found that infertile women suffer economic hardships, their accounts are different. In Fledderjohann’s study, women without children need to work by themselves because they have no child to assist them in times of need. However, in the present study, husbands stop providing for their wives, due to these women’s infertile condition. Some women devised means of survival - work. They were questioned by their counterparts why they worked because, according to these counterparts, women worked for their children.

According to some women, the least confrontation reminded them of their childlessness. They were sometimes victims of circumstances they knew nothing about. Age was no barrier to stigma, consistent with findings in other studies that infertile women are stigmatized throughout their lives [29].

Other women revealed feeling sad or bad when alone or when they heard other children calling their parents. According to them, moments like these reminded them of their failure. They feel they have denied their husbands the experience of fatherhood, a plight echoed in Donkor and Sandall [5].

Notwithstanding, the discomfort and maltreatment from their husbands, some wives preferred to stay in marriage because they feared losing their respect and dignity. This confirms that people marry for different reasons. According to them, they preferred to remain married because of the stigma with divorce [4]. Regardless of the stigma linked to infertility, others stated that their loved ones encouraged and cared about their search for children. For example, some mothers encouraged and advised their daughters to focus on getting children, since children are viewed as a source of happiness, and security for old age and marital stability [8, 32]. This confirms childbirth is essential for a marriage to thrive [8].

Unexpectedly, some infertile women told their friends they had children, just to avoid unnecessary questions and pressure. This finding confirms others’ findings that infertile women pretended to appear fertile to avoid stigma [33] and that such women kept their plight to themselves for fear of others seeing them differently upon disclosure [5]. This finding reveals the intensity of the stigma and how women try to avoid it just to fit into the company of their friends. Consequently, some women felt very uncomfortable around their friends because of the nature of their comments. This also validates earlier findings that some remarks and behaviours of community members were not encouraging to infertile persons [10].

Though married, some women told us they felt having failed as wives, due to their childlessness. This suggests that they have failed to live up to their responsibilities as wives. This result is evident in studies [7] reporting that women are taught right from childhood that they are responsible for childbirth and that,- without children they are incomplete [31].

From the findings, infertile women are deemed worried because of the label given to them by society. Hence, as suggested by Goffman, they are coerced by society to move back and forth giving socially desirable reasons for their predicament making them untruthful and inconsistent [15]. This behaviour was observed among some women when they try to justify reasons for their childlessness. For example, though they were seeking treatment, some revealed they were childless because they were too busy with work.

Again, it was found that the stigma is the main reason for their psychological challenges because they felt unaccepted, side-lined and ignored in their communities. With this, they kept to themselves leading to depression, anxiety and loneliness. Furthermore, some felt they had not lived up to their expectation because society has expectations for married women. Hence, though married, some women still felt they were not wives. Though some wished to leave their marriages, they also feared stigma associated with divorce. It is noteworthy that, due to stigma, victims try to conceal or hide their disabilities. This feature is common among those with hidden disabilities like infertility. However, this becomes impossible because, society has the power to define and project what they deem important and needs attention. This confirms the theory of stigma in a study by Lingsom that disabilities can be visible or invisible although the extent or intensity of its stigma is determined by society [34].

This shows that society is coercive and determines one’s state of being. The plight of these women can be summed up as the combined effects of ‘coercive’ social norms and a prevailing conformism in society.

## Conclusion

From a Darwinian perspective, the survival of the human species depends on reproduction. Infertility is a threat to society and tends to threaten the stability of families and marriage. Our findings show that the burden of infertility or childlessness is aggravated by the reaction from community members, friends, and in-laws. These findings also depict the community is unaware of the effects their reactions have on such women. Hence, the public should be educated on the consequences of stigmatization as well as the need for them to support and encourage infertile women. Structures must be established for continuous sensitization in various communities and sanctions imposed on those found culpable of stigmatizing.

Furthermore, communities, faith-based organisations, and gender-related institutions should hold forums and programmes to sensitize their congregants on marriage and the possible challenges. Counsellors and psychologists are also encouraged to educate would-be couples before marriage on infertility and proffer better approaches to handle such issues. Couples should be educated and devise a plan on how to handle such occurrences.

### Limitations

Future studies should try and balance the socioeconomic and educational background of the sample. In the current study, although the participants were from different socioeconomic backgrounds, most of them were poor and less educated. This could have led to a slight bias in their worldview.

## Data Availability

The data are not publicly available because they contain information that could compromise research participant privacy/consent.
