# Correction to: Living as an infertile woman: the case of southern and northern Ghana

**DOI:** 10.1186/s12978-020-01053-z

**Published:** 2020-12-28

**Authors:** Dorcas Ofosu-Budu, Vilma Hanninen

**Affiliations:** Department of Social Science, University of Eastern Finland, Kuopio, Finland

## Correction to: Reprod Health (2020) 17:69 10.1186/s12978-020-00920-z

Following publication of the original article [1], we have been notified that there is a mistake in the article category mentioned as Review, however, it should be Research article.

## References

[1] Ofosu-Budu D, Hanninen V (2020). Living as an infertile woman: the case of southern and northern Ghana. Reprod Health..

